# Behavioral and psychological factors in individuals with migraine without psychiatric comorbidities

**DOI:** 10.1186/s10194-022-01485-x

**Published:** 2022-08-26

**Authors:** Francesca Pistoia, Federico Salfi, Gennaro Saporito, Raffaele Ornello, Ilaria Frattale, Giulia D’Aurizio, Daniela Tempesta, Michele Ferrara, Simona Sacco

**Affiliations:** Department of Biotechnological and Applied Clinical Sciences, University of L’Aquila, Via Vetoio 1, L’Aquila, Italy

**Keywords:** Migraine, Sleep, Catastrophizing, Anxiety

## Abstract

**Background:**

It is well known that the course of migraine is influenced by comorbidities and that individual psychological characteristics may impact on the disease. Proper identification of psychological factors that are relevant to migraine is important to improve non-pharmacological management. This study aimed at investigating the relationship between psychological factors and migraine in subjects free of psychiatric comorbidities.

**Methods:**

A sample of women with episodic (EM) and chronic migraine (CM) without history of psychiatric comorbidities were included in this cross-sectional study. The study also included female healthy controls (HC) without migraine or other primary headaches. We evaluated sleep, anxiety, depression, intolerance of uncertainty, decision making style and tendence to pain catastrophizing by validated self-report questionnaires or scales. Comparisons among groups were performed using ANOVA and Bonferroni post-hoc tests. Statistical significance was set at *p* < 0.05.

**Results:**

A total of 65 women with EM (mean age ± SD, 43.9 ± 7.2), 65 women with CM (47.7 ± 8.5), and 65 HC (43.5 ± 9.0) were evaluated. In sleep domains, CM patients reported poorer overall sleep quality, more severe sleep disturbances, greater sleep medication use, higher daytime dysfunction, and more severe insomnia symptoms than HC. EM group showed better sleep quality, lower sleep disturbances and sleep medication use than CM. On the other hand, the analysis highlighted more severe daytime dysfunction and insomnia symptoms in EM patients compared to HC. In anxiety and mood domains, CM showed greater trait anxiety and a higher level of general anxiety sensitivity than HC. Specifically, CM participants were more afraid of somatic and cognitive anxiety symptoms than HC. No difference in depression severity emerged. Finally, CM reported a higher pain catastrophizing tendency, more severe feeling of helplessness, and more substantial ruminative thinking than EM and HC, whilst EM participants reported higher scores in the three above-mentioned dimensions than HC. The three groups showed similar decision-making styles, intolerance of uncertainty, and strategies for coping with uncertainty.

**Conclusions:**

Even in individuals without psychiatric comorbidities, specific behavioral and psychological factors are associated with migraine, especially in its chronic form. Proper identification of those factors is important to improve management of migraine through non-pharmacological strategies.

## Background

Migraine is listed among the main causes of disability among people of working age, with a high financial burden linked to direct and indirect costs due to care resource utilization and loss of productivity [1]. Migraine can be distinguished into episodic migraine (EM) with the primary criterion of < 15 headache days per month and in chronic migraine (CM) with ≥ 15 headache days per month. Chronic migraine affects up to 4% of the population: it often develops from EM, through a process named “transformation,” “chronification,” or “progression” [2]. Although recent evidence reports an association between migraine and various comorbidities, the role of coexisting diseases in migraine onset and progression is still unclear [3]. Sleep disorders, depression and anxiety are the comorbidities most frequently reported in association with migraine [4]. A strong bidirectional association between migraine and psychiatric disorders has been systematically documented [5]. It has been reported a significant bidirectional relationship between major depression and migraine, with migraine predicting first-onset depression and depression predicting first-onset migraine [6]. Indeed, a higher prevalence of psychiatric comorbidities has been reported in chronic forms versus episodic forms of headache, especially in CM [5]. Moreover, there is evidence that suicide attempts are more frequent in patients suffering from migraine than in the general population. Shared neurochemical abnormalities between migraine and affective disorders may be the pathophysiologic substrate for their association in susceptible individuals [5].

However, not all the migraine patients have a well-defined history of psychiatric comorbidities. There is a high proportion of patients that report feelings of anxiety, worry and sadness although not showing a clear psychiatric comorbidity or taking treatments for psychiatric illness. This proportion might be increasing in the post Covid-19 era, where subjects with no pre-morbid psychiatric conditions are more likely to experience mental health symptoms resulting in reduced well-being and enhanced psychosocial impairment [7]. Although this issue has not been systematically investigated, empirical evidence suggests that also migraine patients may be badly affected by post-covid psychopathological distress with all the ensuing consequences on migraine-related disability [8].

Overall, poor awareness of specific subtle psychological traits in patients with migraine, even in the absence of psychiatric comorbidities, can negatively affect the overall management of the disease. In recent years, great advances have been made in the pharmacological treatment of migraine, but unmet care needs of patients still remain, due to the poor awareness among medical professionals about the impact of psychological factors on migraine-related disability. Greater attention should be paid to migraine as a bio-psychosocial disorder, where different psychological elements, including anxiety, pain catastrophizing and the tendency to adopt specific decision-making styles based on personality traits, may contribute to the overall disability.

We move from the hypothesis that, even in the lack of well-defined concomitant neuropsychiatric diseases, patients with migraine can show a vulnerable mindset that contributes to a specific migraine phenotype. Specific behavioral and psychological traits are expected to show a different distribution in EM and CM, acting as potential clinical clues for migraine development and progression. According to the main hypothesis, specific behaviors and psychological traits might influence the clinical course of migraine and its progression: specifically, trouble sleeping, dysfunctional personality traits and beliefs, the tendency to pain catastrophizing and to react negatively to uncertain situations, the adoption of specific decision-making styles and the tendency to anxious and hopelessness feelings may identify a vulnerable mindset potentially linked to chronicization. This is in line with the evocative concept of “migraine personality” defined as a mixture of personality features in individuals with migraine including feelings of insecurity with tension manifested as inflexibility, conscientiousness, meticulousness, perfectionism, and resentment [5, 9]. To date, psychological traits, which are likely to influence migraine vulnerability, have not been extensively investigated and little is known whether they are more frequent in chronic forms than in episodic forms.

Therefore, the aim of this study was to investigate the distribution of specific behavioral and psychological factors along the clinical spectrum of migraine and to identify a specific mindset associated with migraine in general, and specifically with CM.

## Methods

### Study population

This was a cross-sectional study performed in a group of patients with episodic migraine (EM) and chronic migraine (CM) and in a group of healthy controls (HC), all without a well-defined history of other neurological or psychiatric disorders ascertained by consulting previous medical records. According to the study design, all the female patients consecutively referring to the Headache Center of L’Aquila/Avezzano in a two-year period with a diagnosis of migraine with or without aura were screened for the inclusion. Migraine diagnosis was performed by experienced neurologists (SS, FP) according to the International Classification of Headache Disorders (ICHD) criteria [10]. Inclusion criteria for patients with EM were: i) age between 30 and 60 years, ii) the fulfillment of ICD-3 criteria for episodic migraine with or without aura, and iii) migraine duration > 5 years. Inclusion criteria for patients with CM were: i) age between 30 and 60 years, ii) the fulfillment of ICD-3 criteria for chronic migraine with or without aura, and iii) no medication overuse. Exclusion criteria for both patient groups were previous or present history of any other type of headache, well-defined history of other neurological or psychiatric disorders, presence of dementia or intellectual disability. Healthy women without migraine were also enrolled in the study as HC and recruited from lists of potential subjects delivered by primary care physicians. Each potential healthy subject underwent an in-person interview with an experienced neurologist (SS, FP) to exclude the presence of migraine or any other primary headache disorder as well as of other chronic pain syndromes. Other exclusion criteria were the same for the migraine and the control group.

The protocol was approved by the Internal Review Board of the University of L’Aquila (02/2017) and an informed written consent was signed by all participants.

### Data collection and neuropsychological assessment

All eligible subjects over the study period were asked to complete a self-report neuropsychological battery with a total administration time of 40 min approximately. The domains investigated by the neurobehavioral battery were sleep, anxiety, depression, intolerance of uncertainty, decision making style and tendence to pain catastrophizing. Sleep quality, insomnia symptoms, and daytime sleepiness were assessed through the Pittsburgh Sleep Quality Index (PSQI), the Insomnia Severity Index (ISI) and the Epworth Sleepiness Scale (ESS) [11–13]: the PSQI assesses sleep quality and disturbances over a 1-month time interval, while ISI quantifies the perceived insomnia severity and ESS provides a measurement of the subject's general level of daytime sleepiness [11–13]. Anxiety symptoms were assessed using the trait-anxiety subscale of the State-Trait Anxiety Inventory (STAI-X2), which investigates the general propensity to be anxious regardless of the situation the person is in that moment, and the Anxiety Sensitivity Index-3 (ASI-3), which measures the fear of symptoms associated with anxiety [14, 15]. The presence of depressive symptoms was assessed by means of the Beck Depression Inventory-second edition (BDI-II), measuring the severity of depression symptoms based on a two-week time period [16]. Moreover, intolerance for uncertainty and ambiguity, and strategies for coping with uncertainty were investigated using the Intolerance of Uncertainty Inventory (IUS-10), the Intolerance of Uncertainty Scale-12 (IUS-12), the Uncertainty Response scale (URS), and the intolerance of ambiguity questionnaire (IA) [17–20]: the IUS 10 and IUS 12 explore the tendency of an individual to consider the possibility of a negative event occurring unacceptable, irrespective of the probability of occurrence [17, 18]. The URS and the IA explore styles of coping with uncertainty and individuals' reactions to ambiguous stimuli and perceived ambiguity [19, 20]. Finally, the decision-making style and the threat value of pain were assessed using the General Decision-Making Style (GDMS) and the Pain Catastrophizing Scale (PCS), respectively [21, 22]: the former evaluates the general cognitive habit influencing the daily decision-making style and the latter explores the presence of exaggerated feelings of rumination, magnification, and helplessness resulting in maladaptive pain coping strategies [21, 22].

### Statistical analysis

The analyses were performed using JAMOVI 1.6 (The Jamovi project, 2020). To investigate putative differences between the three participant groups, total scores of each questionnaire and the respective subscales were separately submitted to an analysis of variance (ANOVA) with the experimental “group” (chronic migraine, episodic migraine, control) as a three-level between-subjects factor. For significant effects, Bonferroni post hoc tests were performed. All tests were two-tailed and statistical significance was set to *p* < 0.05. Effect size estimates were reported using partial eta-square (η^2^_p_) for the ANOVAs [23]. Control analyses including “age” as covariate were performed, confirming all the significant main and post hoc effects.

## Results

Overall, 250 patients (75 CM and 175 EM) met the inclusion criteria and were invited to be included in the study. Out of them, 206 patients (73 CM and 133 EM) provided their informed consent to participate. Seventeen patients with EM and 8 patients with CM were later excluded as they did not have enough time to complete the whole survey. A final set of 181 patients (i.e., 65 CM, 36% of the sample and 116 EM 64% of the sample) completed the self-report neuropsychological battery. As the study hypothesis was a different distribution of behavioral factors in episodic vs. chronic migraine patients, we sub-grouped the whole patient sample comparing EM patients (*n* = 65; mean age ± SD 43.9 ± 7.2) with CM patients (*n* = 65; mean age ± SD 47.7 ± 8.5). EM patients were selected based on age distribution. The HC group was formed by 65 healthy women (mean age ± SD 43.5±9.0) fulfilling the same inclusion criteria.

### Sleep variables

**Fig. 1:**
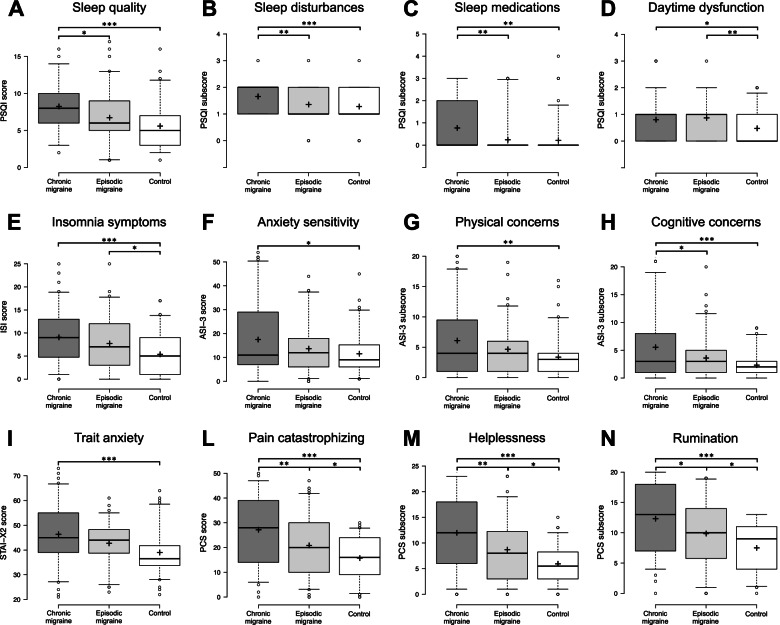
Comparisons between the three groups (chronic migraine, episodic migraine, control) on **(A)** sleep quality (PSQI total score), **(B)** sleep disturbances (PSQI subscale score), **(C)** use of sleep medications (PSQI subscale score), **(D)** sleep-related daytime dysfunction (PSQI subscale score), **(E)** severity of insomnia symptoms (ISI total score), **(F)** anxiety sensitivity (ASI-3 total score), **(G)** concerns about physical anxiety symptoms (ASI-3 subscale score), **(H)** concerns about cognitive anxiety symptoms (ASI-3 subscale score), **(I)** trait anxiety (STAI-X2 total score), **(L)** pain catastrophizing (PCS total score), **(M)** pain-related feelings of helplessness (PCS subscale score), and **(N)** pain-related ruminative thinking (PCS subscale score). Center lines show the medians, box limits indicate the 25th and 75th percentiles, whiskers extend to 5th and 95th percentiles, dots represent outliers, and crosses represent sample means. Asterisks indicate significant Bonferroni post hoc (*p < .05, **p < .01, ***p < .001). *Abbreviations*: PSQI, Pittsburgh Sleep Quality Index; ISI, Insomnia Severity Index; ASI-3, Anxiety Sensitivity Index-3; STAI-X2, trait-anxiety subscale of the State-Trait Anxiety Inventory; PCS, Pain Catastrophizing Scale.

As reported in the Table, the ANOVAs showed a significant effect of the “group” factor for PSQI and ISI total score and some PSQI sub-components (sleep disturbances, sleep medication, daytime dysfunction). Post hoc comparisons (Fig. 1A–E) revealed that CM group was characterized by poorer overall sleep quality (p < 0.001), more severe sleep disturbances (*p* < 0.001), greater sleep medication use (*p* = 0.004), higher level of daytime dysfunction (*p* = 0.034), and more severe insomnia symptoms (*p* < 0.001) as compared to the HC group. EM group showed better sleep quality (*p* = 0.040), lower sleep disturbances (*p* = 0.008) and sleep medication use (*p* = 0.007) than the CM group. On the other hand, post hoc comparisons highlighted more severe daytime dysfunction (0.007) and insomnia symptoms (*p* = 0.047) in EM participants as compared to the HC group.

### Anxiety and depression variables

The ANOVAs revealed significant differences between the three experimental groups on ASI-3 and STAI-X2 total score, and some ASI-3 sub-components (physical concerns, cognitive concerns). Post hoc comparisons (Fig. 1F–I) indicated that the CM group showed greater trait anxiety (*p* < 0.001) and a higher level of general anxiety sensitivity (*p* = 0.018) as compared to the HC group. Specifically, CM participants were more afraid of somatic (*p* = 0.003) and cognitive anxiety symptoms (*p* < 0.001) when compared to HC subjects. Finally, sensitivity to cognitive anxiety symptoms was higher in CM than in EM group (*p* = 0.047). Finally, no difference in BDI-II scores between experimental groups was highlighted.

### Uncertainty variables

Analyses of IUI-10, IUS-12, IA, and URS total score and sub-components did not show any significant effect of “group” factor (see Table 1). Therefore, the three experimental groups did not differ in intolerance of uncertainty and ambiguity, as well as in strategies for coping with uncertainty.

**Table 1:** Descriptive statistics [mean (*SD*)] of questionnaire scores assessing sleep features (PSQI total score and sub-components, ISI, ESS), psychological status (ASI-3 total score and sub-components, STAI-X2, BDI-II), management of uncertainty/ambiguity (IUI-10, IUS-12, IA, URS total score and sub-components), pain catastrophizing tendency (PCS total score and sub-components), and decision making style (GDMS total score and sub-components) for the three participant groups (chronic migraine, episodic migraine, control). Results of ANOVA are also reported (F, p, η^2^_p_)

	**Chronic migraine**	**Episodic migraine**	**Control**			
*Sleep variables*	Mean (SD)			F	p	η^2^_p_
PSQI score	8.25 (3.37)	6.73 (3.59)	5.59 (3.15)	9.87	** < 0.001**	0.096
PSQI subscale score						
Subjective sleep quality	1.41 (0.78)	1.36 (0.81)	1.14 (0.81)	2.04	0.13	0.022
Sleep latency	1.34 (0.95)	1.27 (0.96)	0.97 (0.95)	2.79	0.06	0.029
Sleep duration	1.18 (0.79)	0.98 (0.86)	0.94 (0.73)	1.64	0.20	0.017
Habitual sleep efficiency	1.00 (1.16)	0.72 (1.05)	0.68 (0.95)	1.71	0.18	0.018
Sleep disturbances	1.66 (0.51)	1.36 (0.57)	1.28 (0.55)	8.38	** < 0.001**	0.083
Sleep medications	0.77 (1.23)	0.24 (0.78)	0.22 (0.76)	6.80	**0.001**	0.068
Daytime dysfunction	0.80 (0.81)	0.87 (0.71)	0.48 (0.61)	5.53	**0.005**	0.056
ISI score	9.06 (5.91)	7.71 (5.87)	5.37 (4.53)	7.53	** < 0.001**	0.073
ESS score	5.92 (4.91)	6.15 (3.67)	5.25 (3.68)	0.85	0.43	0.009
*Anxiety and depression variables*						
ASI-3 score	17.48 (15.70)	13.66 (10.50)	11.54 (8.76)	3.98	**0.02**	0.040
ASI-3 subscale score						
Physical concerns	6.10 (5.86)	4.66 (4.15)	3.36 (3.51)	5.60	**0.004**	0.056
Cognitive concerns	5.54 (6.34)	3.60 (3.96)	2.31 (2.30)	8.28	** < 0.001**	0.081
Social concerns	5.84 (5.08)	5.25 (4.45)	5.89 (4.69)	0.37	0.69	0.004
STAI-X2 score	46.37 (12.24)	42.70 (8.58)	38.97 (9.30)	8.43	** < 0.001**	0.082
BDI-II score	11.73 (9.92)	9.54 (7.50)	10.19 (10.63)	0.91	0.40	0.009
*Uncertainty variables*						
IUI-10 score	25.16 (11.17)	25.03 (8.79)	24.14 (9.38)	0.20	0.82	0.002
IUS-12 score	27.35 (11.62)	29.09 (9.48)	27.69 (11.37)	0.47	0.63	0.005
IA score	42.24 (11.43)	40.81 (12.93)	38.46 (13.04)	1.49	0.23	0.016
URS score	123.72 (18.27)	121.30 (16.31)	125.85 (17.62)	1.10	0.34	0.011
URS subscale score						
Emotional uncertainty	31.58 (10.14)	30.58 (8.93)	29.76 (8.68)	0.63	0.54	0.007
Desire for change	44.17 (8.94)	44.56 (8.94)	46.26 (8.21)	1.01	0.37	0.011
Cognitive uncertainty	47.97 (8.98)	46.16 (8.59)	49.05 (9.05)	1.74	0.18	0.018
*Pain catastrophizing variables*						
PCS score	27.18 (13.67)	20.84 (12.13)	15.73 (8.38)	15.32	** < 0.001**	0.141
PCS subscale score						
Helplessness	11.98 (6.99)	8.69 (6.14)	5.94 (3.68)	17.28	** < 0.001**	0.157
Rumination	12.31 (5.69)	9.86 (5.46)	7.50 (3.89)	14.09	** < 0.001**	0.132
Magnification	2.90 (2.19)	2.30 (2.01)	2.25 (1.82)	2.03	0.13	0.021
*Decision making variables*						
GDMS score	78.42 (9.25)	77.55 (10.50)	76.37 (10.77)	0.56	0.57	0.007
GDMS subscale score						
Rational	17.21 (2.23)	16.89 (2.46)	17.04 (2.32)	0.25	0.78	0.003
Intuitive	17.89 (2.99)	17.75 (2.85)	17.21 (3.03)	0.81	0.45	0.010
Dependant	16.49 (3.31)	16.58 (3.58)	16.47 (3.81)	0.02	0.99	< 0.001
Avoidant	13.43 (2.94)	13.16 (2.77)	12.75 (3.05)	0.76	0.47	0.009
Spontaneous	13.40 (2.91)	13.16 (2.73)	12.90 (2.79)	0.44	0.65	0.005

### Pain catastrophizing variables

The ANOVAs revealed a significant effect of “group” factor for PCS total score, and the sub-scales of helplessness and rumination. Post hoc comparisons (Fig. 1L–N) showed a higher pain catastrophizing tendency, a more severe feeling of helplessness, and more substantial ruminative thinking in the CM group as compared to the EM group (*p* = 0.003; *p* = 0.002; *p* = 0.007; respectively) and HC (all *p* < 0.001). EM participants reported higher scores in all the three abovementioned dimensions compared to HC (*p* = 0.013; *p* = 0.007; *p* = 0.009; respectively).

### Decision making variables

No differences in GDMS total score and in the respective subscales were highlighted by ANOVAs.

The three experimental groups showed similar decision-making styles on all domains addressed (rational, intuitive, dependent, avoidant, spontaneous).

## Discussion

Our findings show that a mindset towards anxiety, poor sleeping and catastrophizing is more commonly recognized in individuals with migraine than in healthy subjects. While each of these three conditions are associated to both EM and CM, the association with CM is undoubtedly more evident. In fact, scores of EM participants were in between those recognized in the healthy condition and in CM, thus suggesting a gradient distribution across the explored groups. It is important to highlight that the subjects included in our study were selected based on the lack of concomitant well-known psychiatric comorbidities according to the DSM (Diagnostic and Statistical Manual of Mental Disorders)-V [24]. This choice aimed at identifying a vulnerable psychological profile of individuals with migraine, even in the lack of diagnosed mental health disorders. In fact, while the association of migraine with specific major psychiatric diseases is well-recognized, less is known about the influence of individual psychosocial factors on the migraine brain of otherwise healthy subjects with a lower mental health resilience. Our study makes a step forward in identifying which behavioral and psychological factors may increase migraine vulnerability in individuals not showing any mental health issues. The domains explored in this study included the sleeping behavior, the affective dimension, the tendency to experience unknown outcomes as unacceptably threatening, the decision-making style and the tendency to pain catastrophizing. The finding that poor sleeping, anxiety feelings and catastrophizing thinking are more likely to be found in association with migraine prompts reflections on the connotation of migraine as a vulnerable brain state, that can be influenced by daily behavioral and psychosocial stressors. In the same venue, another study in a sample of patients with chronic migraine and affective temperamental dysregulation interestingly found a relationship between the degree of temperamental dysregulation and the presence of suicidal ideation, feelings of hopelessness and negative attitudes about the future, albeit in the absence of previous diagnosed psychiatric disorders [25]. This suggests a possible predictive value of affective temperamental dysregulation for migraine chronicization and patient’s clinical outcomes.

Although the influence of behavioral factors on migraine is not fully understood, a growing body of evidence suggests that the interaction of biological, sociocultural and psychological elements can influence the migraine’s characteristics. Migraine is a biological entity with underlying mechanisms targeted by symptomatic and preventive treatments that are constantly evolving and provide patients with ever better opportunities to manage the disease. However, for a significant minority of patients there is a limited benefit with pharmacotherapy alone [26]. A possible reason for this is the presence of specific psychological traits that, even in the absence of major psychiatric diseases, contribute to a vicious cycle where maladaptive behavior and migraine feed off each other [26]. This is in line with recent data suggesting that, although monoclonal antibodies targeting calcitonin gene-related peptide (CGRP) pathway are usually effective in difficult-to-treat patients, the presence of “anxious-fearful” personality together with current stressors and anxiety represent negative predictors of treatment outcome [27]. All these observations suggest that a purely biomedical model of migraine is not fully appropriate to understand the various aspects of the disease and to improve the quality of life of patients. A biopsychosocial model of migraine could pave the way for an alternative management approach, by guiding clinicians to combine pharmacological treatments, behavioral interventions, and stress management strategies to reduce migraine-related disability. Within this framework, non-pharmacological interventions have not to be considered alternative to standard headache care, but as an additional treatment option for all the patients.

Indeed, whether migraine precedes psychological changes or it is triggered and exacerbated by them is still debated: the two conditions certainly feed each other in a vicious circle that reduce the patient’s well-being, with a growing demand for drugs acting both at pain and at behavioral level. Moreover, the tendency of patients with migraine to experience poor sleep quality, greater anxiety sensitivity and pain catastrophizing, associated with feelings of hopelessness and rumination behaviors, suggests a reciprocal influence between migraine and behavioral changes.

Perceived stress has received particular attention as a trigger factor for headache both in episodic and chronic forms [28, 29]. Stress can be defined as an organism’s perception of and response to a perceived stressor [28, 29]. This includes how the body responds to perceived threats, challenges, or physical or psychological barriers, by activating the autonomic nervous system, especially through the hypothalamic-pituitary- adrenal (HPA) axis [29]. The individual response becomes dysfunctional when the stress pressure exceeds one’s perceived ability to cope with stress itself: this occurs when subjects have dysfunctional thoughts that feed a vicious feedback cycle potentially amplifying the pain related disability [29]. In physiologic conditions, when individuals experience a stressful event, the mechanism of allostasis usually induces self-limiting responses that revert to a normal baseline status when the individual withdraws from the external stress or challenge. When an hyperactivation of the allostatic system occurs, due to chronic stressors or underlying dysfunctional thoughts, a pathophysiologic response, named allostatic load or overload, interferes with the neuroendocrine, cardiovascular, immune, and metabolic homeostasis and results in pathophysiologic conditions associated with chronic diseases [29]. While allostasis contributes, together with other homeostatic mechanisms, to stress adaptation, the allostatic load represents the cumulative effect of chronic physiologic stress, which may be generated by internal processes or external factors. Internal processes include the tendency to develop anxiety feelings and catastrophizing thinking, while external factors are represented by chronic stressors or lifestyle habits such as poor sleep hygiene. Many studies suggest that an unfavorable allostatic load may contribute to the new onset of migraine, although a direct causal relationship with stress is still debated [28, 29]. Moreover, major stressful events, especially in vulnerable individuals, can be associated with migraine chronification, in a view of reciprocal influences between pain and behavioral dysfunctional states. Indeed, although stress is the most reported self-identified trigger for migraine, the sudden decline of perceived stress has also been reported as a precipitating factor for the onset of migraine attacks, especially with respect to “weekend”, “honey-moon” and “let-down” migraines [30–32]. These observations are in line with data from studies investigating the co-occurrence of migraine and psychopathological symptoms from young age onwards: children with primary headaches, especially with migraine, often have an association with sleep and behavioral disorders and about the half of adolescents with chronic daily headache presents with at least one psychiatric comorbidity, mostly major depression and panic disorder [33, 34]. Adolescents with migraine are more likely to develop a specific psychiatric disorder later in life as compared to adolescents without headache: therefore, specific attention is paid to the period of transition from adolescence to adulthood when a greater tendency to experience anxiety, sleep disorders and catastrophizing feelings can predispose to develop later psychiatric diseases [33, 34]. Non pharmacological interventions may be particularly useful at this age, when resilience‐training programs are more effective. In the adult age, anxiety, depression, post-traumatic stress disorder and sleep disorders are the most frequent comorbidities in subjects with primary headaches, acting as trigger for headache or as risk factors for chronicity [3, 35]. Specifically, anxiety in association with sleep-related disturbances may be a driver for migraine development and transformation due to shared pathophysiological pathways: a polymorphism in the 5-HT transporter gene and a specific dopamine D2 receptor genotype, an imbalance of serotonin neurotransmitters as well as a disproportion between pro-inflammatory and anti-inflammatory cytokines in the hypothalamic-pituitary adrenal axis may account for the simultaneous presence of these comorbidities [3, 35]. Moreover, neurofunctional data from 18 F-fluorodeoxyglucose positron emission tomography recently confirmed that CM patients have a decreased metabolism in prefrontal areas including frontal pole, superior and inferior frontal cortex as well as orbitofrontal areas, that are all structures associated with cognitive, affective, and sensory processing functions [36]. Prefrontal cortex is connected to limbic regions, which can regulate pain modulation in humans, especially in chronic pain conditions. This further endorses the possible bidirectional association between psychiatric disorders and migraine and the need of optimizing the pharmacological and non-pharmacological treatment of both [36].

Strengths of our study include the large sample, the rigorous selection of patients based on predefined criteria, and the comprehensive neuropsychological battery used for assessment. To limit the effect of confounders we included a highly selected sample. The choice to include women only was moved from the assumption that behavioral and psychological factors may operate differently in men and women, so analyzing them separately by gender is advisable. Moreover, we excluded patients beyond 65 years, as cognitive related issues in more advanced ages could have interfered with the proper compilation of the self-report neuropsychological battery. Finally, we excluded patients with medication overuse as commonly showing a more complex behavioral profile which requires separate assessment and analysis as objective for future research.

Limits of the study include the reduced generalizability of our findings to the whole population of migraineurs: in fact, while the use of rigorous criteria for enrolment represents for certain aspects a point of strength, the findings are not transferable to a broader group of people or situations, for instance men or older patients. The other limit is the adoption of a cross-sectional design which does not allow drawing conclusions about a causative relationship between the presence of specific behavioral traits and the development of migraine or its chronification: further longitudinal studies could allow investigating whether the presence of specific psychological traits and maladaptive behaviors really influences migraine progression and chronifications in a causal way. Moreover, self-reported measures may have some limits in evaluating psychosocial dimensions: once self-reported questionnaires have identified a vulnerable mindset for migraine, in-person interviews with the patient would be of help in orientating the overall management.

With these limits, our results suggest that behavioral and psychological factors, even in individuals without psychiatric comorbidities, are associated with the presence of migraine in general and of CM in particular. Further research is needed to address how behavioral and psychosocial issues “get under the skin” and trigger the vicious cycle potentially underlying migraine development and chronification.

Research in this area may have direct implications in clinical practice by providing patients with an integrated therapeutical approach characterized by a high degree of collaboration among different health professionals, also including psychologists. Pharmacotherapy alone is improbable to be paramount in managing migraine in a context of concomitant behavioral dysfunctions. Non-pharmacological treatments, such as acupuncture, electromyographical biofeedback, relaxation training, cognitive behavioral therapy or mindfulness-based approaches, would be essential to provide complementary treatment options enhancing the efficacy of ongoing pharmacologic interventions. Therefore, evidence on the role of psychological and behavioral factors on migraine clinical course needs to be integrated into innovative conceptual frameworks to better organize education and evidence-based clinical practices, in the view of a unified biopsychosocial approach to migraine care. This approach encompasses education and behavioral treatments as well as pharmacologic therapy and may contribute to improve the partnership with the patients and to reduce the burden of migraine, as well as the risk for medication overuse.

## Data Availability

The datasets used and/or analysed during the current study are available from the corresponding author on reasonable request.

## Abbreviations

EM: Episodic migraine
CM: Chronic migraine
HC: Healthy control
